# Horizontally Aggregation of Monolayer Reduced Graphene Oxide Under Deep UV Irradiation in Solution

**DOI:** 10.1186/s11671-019-2940-z

**Published:** 2019-04-02

**Authors:** Xiaoxiao He, Sanjun Zhang, Haifeng Pan, Jinquan Chen, Jianhua Xu

**Affiliations:** 1 State Key Laboratory of Precision Spectroscopy, East China Normal University, Shanghai, 200062 China; 2 Collaborative Innovation Center of Extreme Optics, Shanxi University, Taiyuan, 030006 Shanxi China

**Keywords:** UV exfoliation, Aggregation of rGO, Monolayer, Few layer nano-graphene

## Abstract

Graphene has been widely used in novel optoelectronic devices in decades. Nowadays, fabrication of large size monolayer graphene with spectral selectivity is highly demanded. Here, we report a simple method for synthesizing large size monolayer graphene with chemical functionalized groups in solution. The few layer nano-graphene can be exfoliated into monolayer nano-graphene under short time UV irradiation in protic solution. The exfoliated monolayer nano-graphene could experience deoxygenation during long time UV exposure. At the same time, the edge of nano-graphene could be activated under deep UV exposure and small size nano-graphene sheets further aggregate horizontally in solution. The size of aggregated rGO increase from 40 nm to a maximum of 1 μm. This approach could be one promising cheap method for synthesizing large size monolayer reduced graphene oxide in the future.

## Background

Graphene is a potential material for ultrathin optoelectronic and photodetection devices because of its high carrier mobility and high optical transparency [1, 2]. The key to the high photoresponse of graphene-based devices is the fermi level shifting that induced by the injunction of carriers [3]. With the development of chemical vapor deposition (CVD), growth of large size graphene as well as fabrication of graphene-based devices becomes convenient. However, graphene-based photoresponse device usually has weak absorption and poor spectral selectivity. The common method used to overcome this drawback is hybridizing graphene with quantum dots [4], plasmonic nanostructure [5], or other 2D materials with energy gaps [6] in order to achieve photo-induced carrier injection. Although CVD method promotes the fabrication of growth of large size graphene, the deposition process commonly happens in extreme environment, such as high vacuum, highly selected substrate, and so on. This limits the enlargement fabrication for commercial manufacture. New and low-cost methods are urgent to be developed. Solvent-mediated exfoliation for few layer flakes is one of the efficient and low-cost methods in graphene fabrication [7–15]. The most widely used method is modified Hummer’s method. The graphite can be oxidized and exfoliated into few layer graphene. Meanwhile, graphene fabricated via chemical oxidized exfoliation usually contains various functional groups which can enhance the optical absorption and spectral selectivity. On the other hand, the oxidized exfoliation process usually damages the crystallinity of sp^2^ domain [16], which requires extremely high temperature for recovery. Although the thermal reaction process could recover the sp^2^ domain, almost all the functional groups are also removed, leading to weak absorption and poor spectral selectivity again. Herein, we report a new strategy to fabricate large size chemical functionalized monolayer graphene by deep UV irradiation. The layered nano-graphene can be exfoliated to monolayer under short time UV exposure. The new sp^2^ domain can be restored during long time UV exposure. Furthermore, the edge carbon atom can be activated during UV irradiation, leading several monolayer nano-graphene sheets to aggregate horizontally to form large size monolayer graphene.

## Experimental Method

### Fabrication of Graphene Oxide

Graphene oxide (GO) was synthesized from natural graphite by modifying the Hummer’s method as reported in our previous work [17]. The resulting mixture was washed by 5% HCl solution and DI for dozens of times. Finally, GO solid was obtained after freeze-drying.

### Synthesis of Few Layer Nano-Graphene and Growth for Large Size Reduced Graphene Oxide

4.4 mg GO solid was transferred to Teflon-lined autoclave and 12 mL ethanol (or *N*,*N*-dimethylformamide (DMF)) was added. The mixture was heated to 176 °C for 5 h. The supernatant was filtered through a 0.22-μm microporous membrane. Finally, the colloidal solution was the few layer nano-graphene solution.

4.4 mg GO solid was transferred to Teflon-lined autoclave and 15 mL DI added. The mixture was heated at 176 °C for 5 h. Then the supernatant was filtered through a 0.22-μm microporous membrane. The colloidal solution was the monolayer nano-graphene solution.

**Scheme 1:**
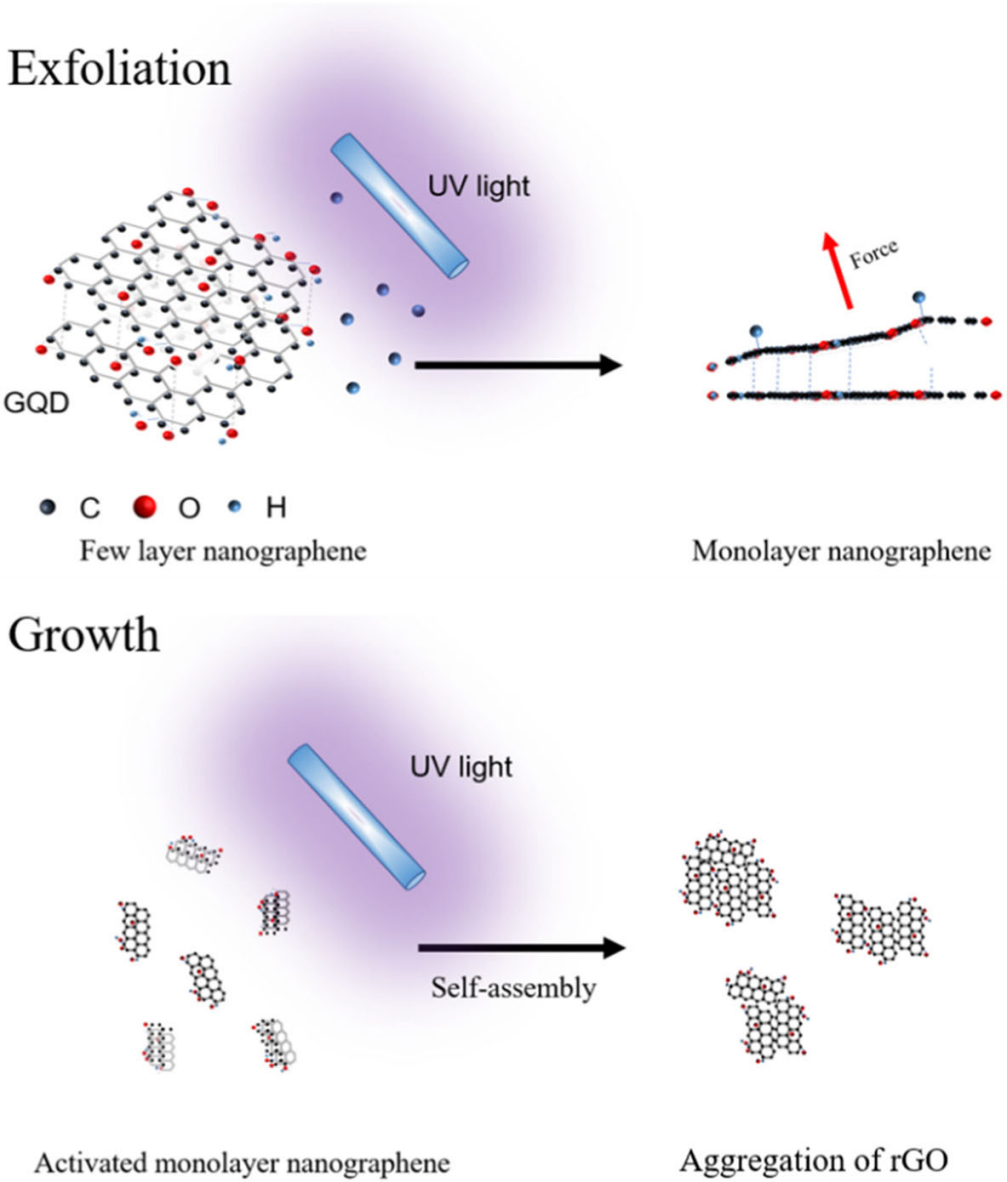
Schematic illustration of exfoliating of few layer nano-graphene into monolayer and aggregation of rGO under UV irradiation

The exfoliation of few layer nano-graphene and growth for large size reduced graphene oxide (rGO) were obtained by deep UV light (3 W, 254 nm) irradiating, as illustrated in Scheme 1.

### Sample Characterization

The photoluminescence (PL) and Fourier-transformed infrared (FTIR) were measured on steady-state fluorescence spectrometer (FluoroMax-4, Horiba, Jobin Yvon) and FTIR spectrometer (Nicolet 8700, Thermo Scientific), respectively. The morphology and height were characterized by atomic force microscopy (AFM) operating in tapping mode at room temperature on Si substrate (NT-MDT Prima). The crystallinity of the sample was performed by high resolution transmission electron microscope (HRTEM) (JEM-2100F, JEOL).

## Result and Discussion

**Fig. 1:**
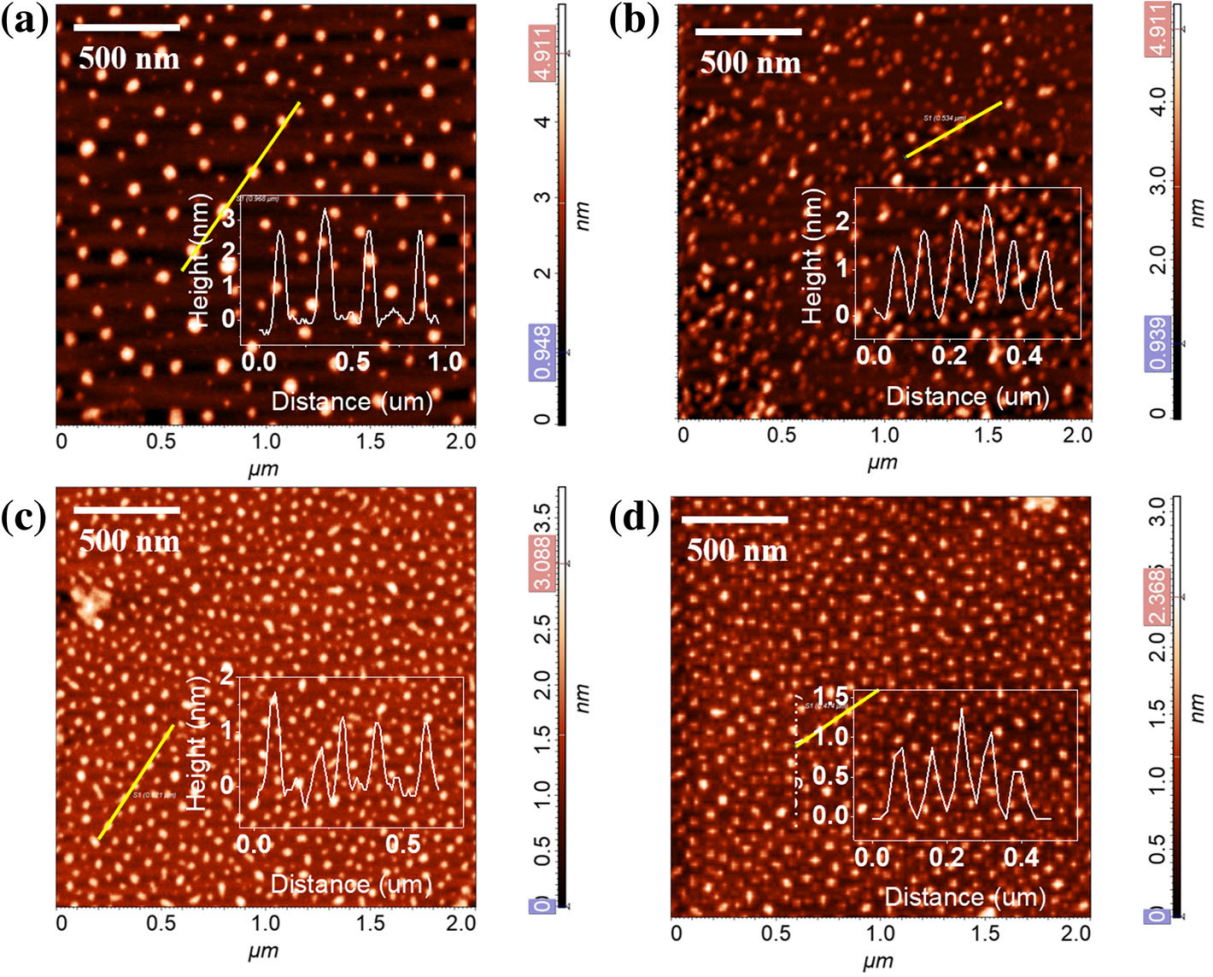
AFM images of few layer nano-graphene with various UV exposure time **a**–**d**: 0, 30 s, 50 s, 240 s, insert is the height distribution of nano-graphene

AFM was used to characterize the morphology and size of nano-graphene. The results of nano-graphene under various UV irradiation time are shown in Fig. 1. For the fresh nano-graphene, the diameters mainly distributed in the range of 30–60 nm and the height is > 2.5 nm (Fig. 1a). The height of our nano-graphene is similar to those observed in chemical group functionalized nano-graphene with 2–3 layers thickness [10, 18, 19]. From AFM data, we determine that more than 85% nano-graphene are formed by stacking 2–3 layers nano-graphene monomer through Van der Waals (vdW) force. Thus, we name them as few layer nano-graphene. The few layer nano-graphene could be exfoliated into monolayer nano-graphene under 254 nm UV lamp irradiation (3 W). Figure 1b–d shows the morphology of few layer nano-graphene under 30 s, 50 s, 240 s UV exposure, respectively. There is barely change in size after short time UV exposure. However, the height distribution for nano-graphene under various UV exposure times clearly show that the height of few layer nano-graphene decrease from > 2.5 nm to < 1.0 nm after several minutes UV exposure, indicating that the few layer nano-graphene have been exfoliated to monolayer ones.

**Fig. 2:**
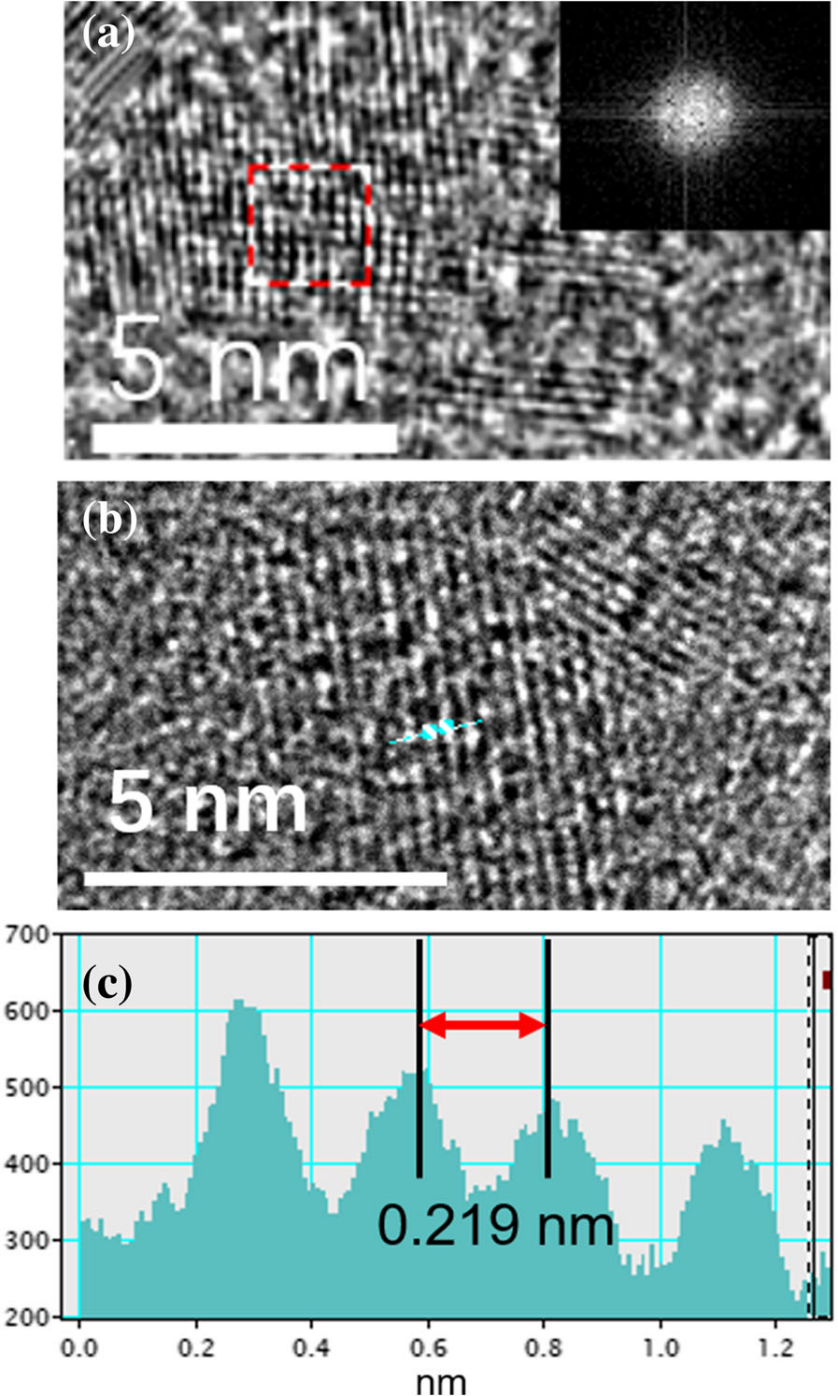
**a** HRTEM images of nano-graphene. Inset: FFT image of a selected area. **b** HRTEM and **c** line profile of the selected nano-graphene

Figure 2a is a high resolution transmission electron microscope (HRTEM) image of monolayer layer nano-graphene prepared by UV irradiation. The crystal lattice of nano-graphene can be clearly observed. The Fast Fourier Transform (FFT) of the selected region is shown in the inset of Fig. 2a, reflecting the hexagonal crystal structure. The in-plane lattice spacing is 0.219 nm, which is consistent with the lattice of the [001] plane [20].

**Fig. 3:**
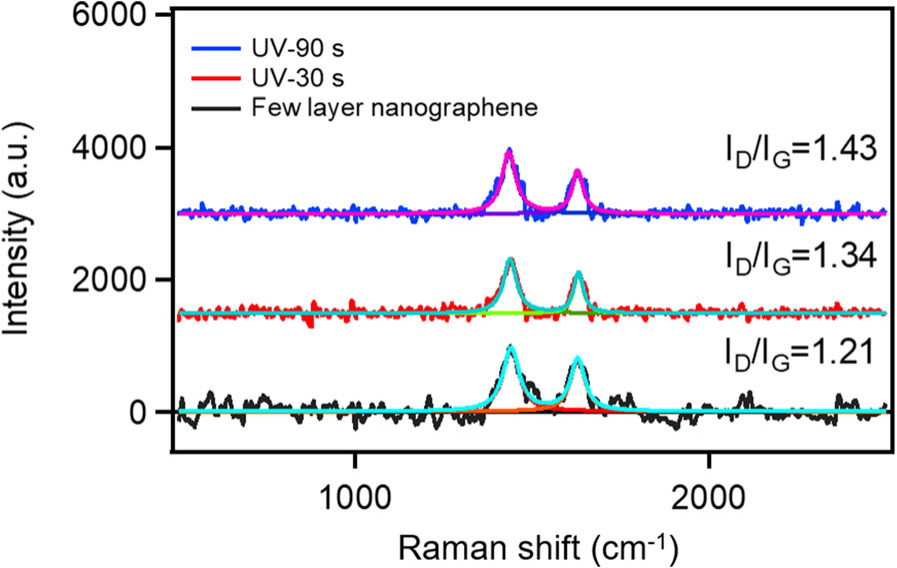
Raman spectra of few layer nano-graphene under various UV exposure time

As reported by Lee et al. [21], hydrogen atoms bind to the surface of graphene and they can bring carbon (C) atoms an out-of-plane force. This force changes the configuration of the carbon atoms in the graphene and it can destroy the vdW force between different graphene layers. Therefore, under the action of hydrogen bonding, the stacked graphene will be exfoliated to a single layer. According to the literature, Raman spectroscopy of graphene can provide important information for planar hydrogenation in graphene [21, 22]. The intensity ratio between *D* and *G* (*I*_*D*_/*I*_*G*_) model of Raman scattering can reflect how hydrogen atom is attached to the graphene. To investigate the exfoliation mechanism, the Raman spectra of few layer nano-graphene under various UV exposure times were measured. The results are shown in Fig. 3. The Raman spectra were fitted by Lorenz model to accurately calculate the peak intensity ratio. The data shows that *I*_*D*_/*I*_*G*_ increases from 1.21 to 1.43, indicating the presence of binding hydrogen on the few layer nano-graphene surface after UV exposure. C atom moves toward the out-plane direction and lead to breaking of the vdW force between graphene layers [21]. As illustrated in Scheme 1, hydrogen atom can bind on the surface of few layer nano-graphene and the out-of-plant force induced by rearrange of C atom can exfoliate the few layer nano-graphene to monolayer nano-graphene.

**Fig. 4:**
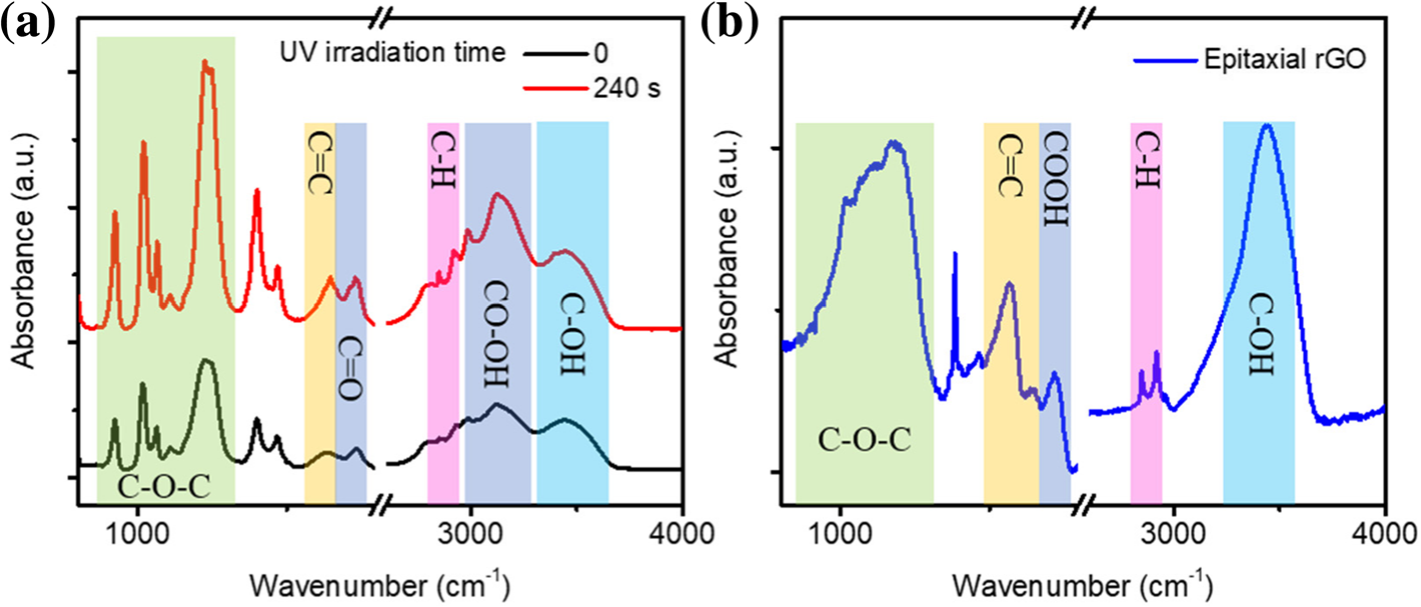
FTIR of (**a**) few layer nano-graphene (black line), monolayer nano-graphene (red line), and (**b)** epitaxial nano-graphene

The surface of the graphene oxide is generally rich in oxygen-containing functional groups such as -COOH, C-OH, C-O-C, and so on. These oxygen-containing functional groups introduce defect states, which are important sources of graphene oxide luminescence. Therefore, it is very important to study the changes of the functional group of nano-graphene during UV irradiation. Infrared absorption is a very effective method for studying chemical functional group changes. The exfoliation mechanism can be more deeply understood by measuring the changes in the infrared absorption spectra of the nano-graphene irradiated by ultraviolet light. Figure 4 shows the Fourier-transform infrared (FTIR) spectra of few layer nano-graphene under different UV irradiation time. For the few layer nano-graphene, vibrational modes are shown for epoxides (C-O-C, 900–1260 cm^−1^). The absorption peak at 1740 cm^−1^ and 3129 cm^−1^ correspond to the stretching mode of C atom bonded carboxyl (-COOH). The absorption at 2850 cm^-1^ and 2920 cm^−1^ reveal the existence of C-H. The broad band absorption (3100–3700 cm^−1^) peaked at 3450 cm^−1^ is attributed to the hydroxyl groups (-C-OH). The C=C absorption of intrinsic sp^2^ domain at 1641 cm^−1^ is also presented. After a short time (240 s) UV irradiation, the peaks of epoxy group, hydroxyl group, carboxyl group, and C=C did not change significantly. However, the absorption peak of C-H becomes more pronounced. This is consistent with the result we obtained from Raman, indicating that the H atom binds to the C atom and enhances the absorption of C-H. It also shows that short-time UV irradiation does not lead to the reduction of nano-graphene and oxygen functional groups in nano-graphene do not change during the exfoliating process.

On the other hand, the FTIR spectra of nano-graphene changes significantly (Fig. 4b) after a long period of UV irradiation (2 h). The first change is that absorption of -COOH at 3150 cm^−1^ is significantly reduced. At the same time, new C-O-C absorption occurs and it overlaps with previous C-O-C absorption, resulting in extensive C-O-C absorption. Secondly, absorption of C=O moves from 1740 cm^−1^ to low wavenumber direction (1720 cm^−1^). This is due to the increase of the conjugate system. The third major change is the appearance of a new C=C in-plane absorption peak at 1562 cm^−1^. This is because that photo-reduction process of GO can induce deoxygenation and restore the sp^2^ domain [16, 23, 24]. Finally, a further enhancement of C-H absorption is observed since more H atoms combine with C atoms.

**Fig. 5:**
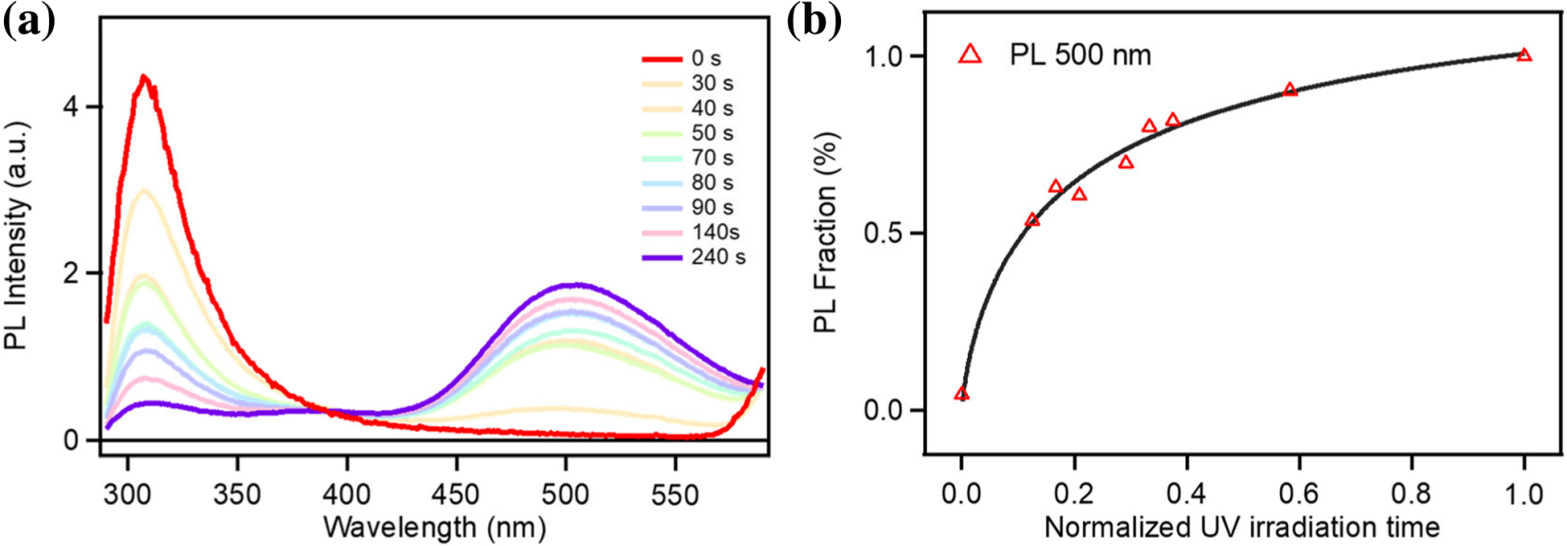
Steady PL spectra of few layer nano-graphene with various UV exposure time excited at 270 nm

In order to study the effect of layer changes on the optical proprieties, we measure the steady-state fluorescence of nano-graphene under different UV exposure time. Figure 5a shows the PL spectra of nano-graphene under various time UV irradiation. The as-prepared few layer nano-graphene emit pure deep UV light peaked at 307 nm and the intensity rapidly decrease with increasing UV irradiation time. Meanwhile, the PL intensity centered at 500 nm increases. The curves in Fig. 5b present the relationship between UV irradiation times and the PL intensity at 307 nm and 500 nm. As the UV irradiation time increases, the 307 nm emission almost disappears, and the 500 nm visible light emission dominates the PL spectrum during the exfoliation process.

Fluorescence of graphene has been systematically studied. Origin of luminescence is mainly assigned to eigenstate-induced fluorescence caused by the sp^2^ domain (302–380 nm) and sp^3^ defect luminescence caused by the oxygen-containing functional group [19, 25–27]. For the few layer nano-graphene, the Van der Waals (vdW) heterojunction forms due to the stacking of few layers nano-graphene. The vdW heterojunction performs high charge separation. The excited electron in surface defects states induced by oxygen functional groups would transfer to intrinsic states induced by C=C sp^2^ domain due to the stacking induced band bending. The few layer nano-graphene emit pure UV light. We assign luminescence with peak at 307 nm to the fluorescence of the eigenstate of the sp^2^ domain. Meanwhile, the visible portion (peak at 500 nm) is derived from the luminescence of the defect states as literature reports [19, 25]. It is clear that fluorescence of the eigenstate of the sp^2^ domain gradually disappears as the nano-graphene is exfoliated into monolayer and we believe that thickness change of nano-graphene is the main reason for the fluorescence change.

**Fig. 6:**
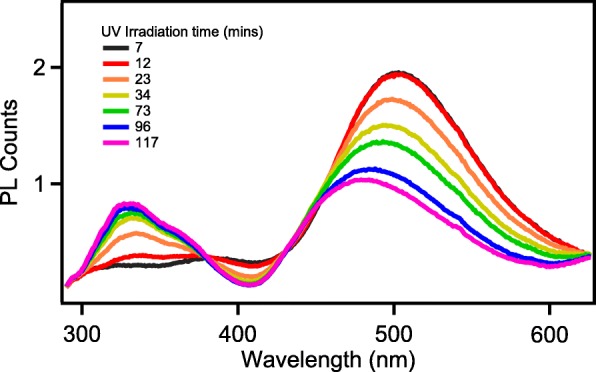
Steady PL of monolayer nano-graphene after different UV exposure periods

For a clearer, intuitive representation of the change in graphene oxide functional groups during the reduction process, we measured steady-state fluorescence during the reduction process (Fig. 6). The fluorescence of the visible portion of nano-graphene is derived from surface defects caused by oxygen-containing functional groups. Different functional groups cause different depth of defect states, which also lead to different fluorescence emission [26, 27]. Konkena et al. studied in detail the relationship between functional groups and fluorescence. The fluorescence of our nano-graphene at 500 nm is derived from the deprotonated carboxyl group. As shown in Fig. 6, increasing the UV exposure time leads to a gradual decrease in the PL intensity at 500 nm and the emission peak shifts from 500 nm to approximately 475 nm after 2 h UV exposure. The evolution of spectra in visible range is similar to that reported in chemical reduction process of graphene [16, 28]. The characteristic emission at 500 nm is the symbol of carboxylic groups in their deprotonation states. The blue (475 nm) emission originates from the dissociated carboxylic groups [28]. With increasing the UV irradiation time, the carboxylic groups were dissociated, which is in accordance with the vanishing of IR absorption of C=O at 1730 cm^−1^ (Fig. 4). Thus, the PL spectra perform decrease and blue shift in visible emission.

Accompanied with the visible emission change, a surprising phenomenon, UV emission centered at 326 nm with a shoulder at 360 nm is shown and increase with reduction time. The reduction of graphene oxide is usually accompanied by the formation of a new sp^2^ domain [29]. We speculate that it may be due to the aggregation of nano-graphene causing an increase in the ultraviolet fluorescence.

**Fig. 7:**
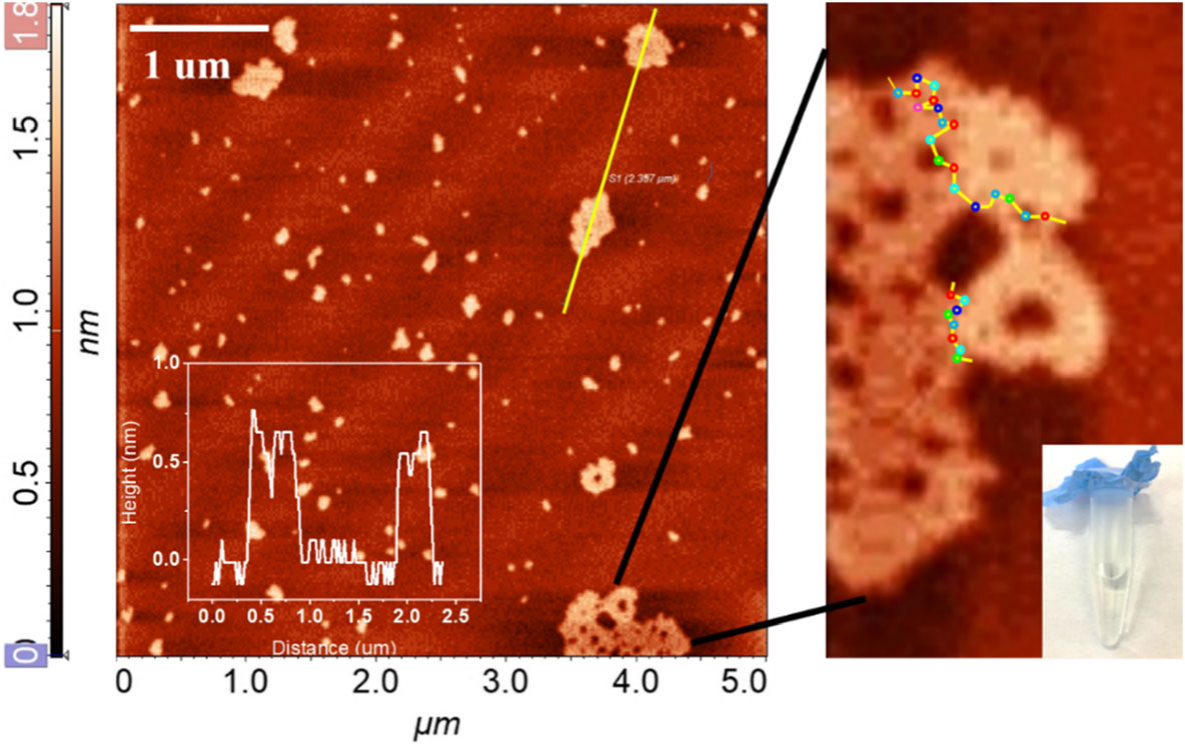
AFM of monolayer nano-graphene under excess UV exposure, inset: digital photos of aggregated rGO

To further examine the changes of physical structure for nano-graphene after long time UV irradiation, we measured the morphology of rGO using AFM. As illustrated in Fig. 7, many large size nanosheets were observed after 2 h UV exposure. The size distribution of rGO is much wider (300–750 nm) compared with that of as-prepared nano-graphene. The increased size can be attributed from horizontal aggregation of monolayer nano-graphene. The enlarge AFM image clearly shows that the larger nano-sheets is surrounded by many small size nano-graphene sheets with 0.7 nm average height. After deoxygenation process, the O atoms at the edge of nano-graphene are active and could bond with the edge C atom of another nano-graphene to form new epoxides. As illustrated in Scheme 1, with increasing reduction, the number of activated nano-graphene increase and the size would continue increase. Considering that as the UV irradiation time is extended, the ultraviolet light-emitting portion is enhanced. We believe that UV luminescence at 326 nm comes from the aggregation of nano-graphene. As the number of agglomerates increases, the intensity of ultraviolet fluorescence also increases. The monolayer nano-graphene is stable and no precipitate was seen even after 2 h UV irradiation (Fig. 7).

**Fig. 8:**
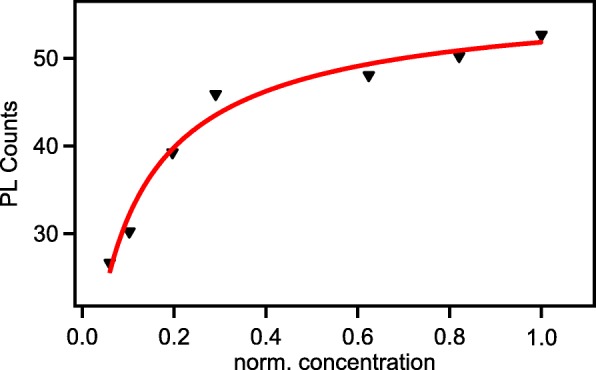
UV PL intensity vs. normalized concentration

Because the intensity is directly related to the aggregation, we investigate the aggregation dynamics of nano-graphene by analyzing the PL in the UV range. The UV exposure time was normalized for the concentration of nano-graphene bonded to each other. Figure 8 presents the concentration correlation with UV intensity. The experimental data were fitted by Langmuir adsorption isotherm model. With increase of UV exposure time, the number of aggregated nano-graphene also increases as well as the size increasing. The number of adsorbed nano-graphene can be expressed as *N* and the Langmuir adsorption isotherm can be written as

1 $$ N=\frac{N_0k{(nx)}^{\left(1-c\right)}}{1+k{(nx)}^{\left(1-c\right)}} $$

Here, *N*_*0*_ is the initial UV intensity. The associated equilibrium constant *k* can be obtained by fitting the experimental data. For a system at chemical equilibrium, the Gibbs free energy is given

2 $$ \Delta G=- RT1\mathrm{n}(k) $$

When we applied *k* to the Eq. 2, we yield the Gibbs free energy *∆G* ≈ − 4.43 kJ/mol. The *∆G* < 0 indicates that this reaction is thermodynamics favored at room temperature [30]. Thus, several number of nano-graphene could bond together horizontally to form large size agglomerates. It is worth to point out that large size graphene prepared via CVD owns fine sp^2^ structure but hardly contains any functional groups for spectral selectivity. Our UV induced nano-graphene aggregation could contain various functional groups which have unique optical property and can be used as identification sites for selective detection.

**Fig. 9:**
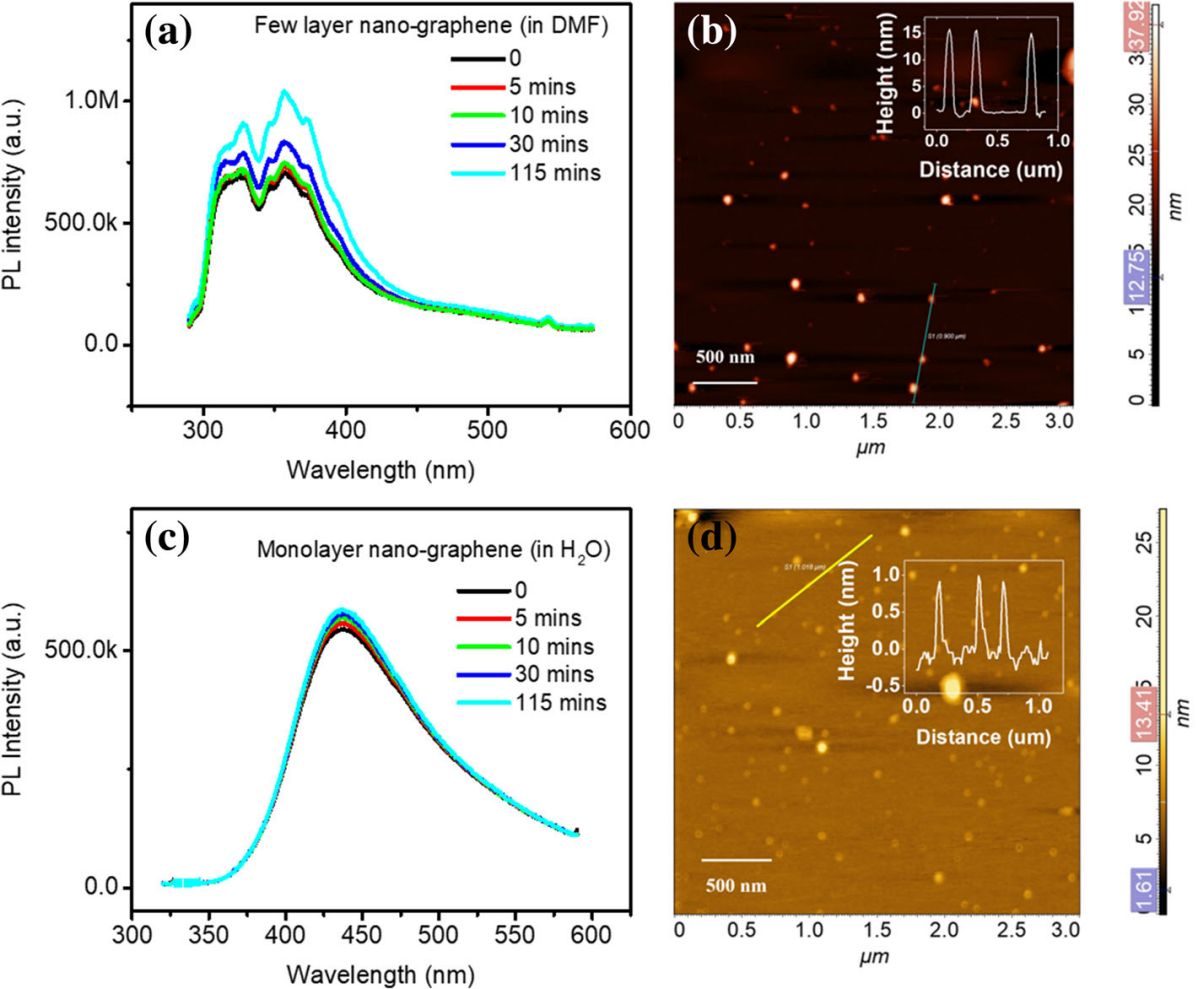
PL and AFM image of nano-graphene in DMF (**a**, **b**) and H_2_O (**c**, **d**)

In order to study the effect of solvents on the structure of nano-graphene, we prepared nano-graphene in aprotic solvent DMF and protic solvent H_2_O. Figure 9 shows the PL and AFM of nano-graphene fabricated in DMF and H_2_O. The PL of nano-graphene in DMF performs deep UV emission, which origin from the stacking of nano-graphene layer. There is only slight change even after 115-min UV irradiation. It indicates that after a long period of exposure to ultraviolet light, no exfoliation or aggregation happens. The height distribution of nano-graphene in DMF is shown in Fig. 9b. The average height is > 15 nm. The PL of nano-graphene in H_2_O is centered at ~ 475 nm (Fig. 9c). There is UV emission and peak shift under various time UV irradiation. This visible emission of nano-graphene is attributed to oxygen functional induced defects states. The height of nano-graphene in H_2_O is < 1.0 nm (Fig. 9d) and it suggest that the nano-graphene is monolayer in H_2_O. The results above show that stacking of nano-graphene can be controlled by using different solvents.

## Conclusions

In summary, we discovered that few layer nano-graphene can be exfoliated to monolayer nano-graphene due to the bonded H atom on the surface under short time deep UV irradiation in protic solution. The monolayer nano-graphene could aggregate into large size monolayer rGO under excess time UV irradiation. The AFM results clearly show that large size monolayer rGO is formed by aggregation of several small nano-graphene sheets. The aggregation of small nano-graphene agrees with the Langmuir adsorption isotherm model, which indicates that the edge of nano-graphene can be activated and can bond with other nano-graphene. This UV-induced growth method may promote the low-cost, large-scale fabrication for monolayer graphene in the future.

## Abbreviations

AFM: Atomic force microscopy
CVD: Chemical vapor deposition
FTIR: Fourier-transform infrared
GO: Graphene oxide
PL: Photoluminescence
TEM: Transmission electron microscope
